# Dynamical Conventional Neural Network Channel Pruning by Genetic Wavelet Channel Search for Image Classification

**DOI:** 10.3389/fncom.2021.760554

**Published:** 2021-10-27

**Authors:** Lin Chen, Saijun Gong, Xiaoyu Shi, Mingsheng Shang

**Affiliations:** ^1^Chongqing Institute of Green and Intelligent Technology, Chinese Academy of Sciences (CAS), Chongqing, China; ^2^School of Information Science and Technology, Tibet University, Lhasa, China

**Keywords:** neural network pruning, neural architecture search, wavelet features, neural network compression, image classification

## Abstract

Neural network pruning is critical to alleviating the high computational cost of deep neural networks on resource-limited devices. Conventional network pruning methods compress the network based on the hand-crafted rules with a pre-defined pruning ratio (PR), which fails to consider the variety of channels among different layers, thus, resulting in a sub-optimal pruned model. To alleviate this issue, this study proposes a genetic wavelet channel search (GWCS) based pruning framework, where the pruning process is modeled as a multi-stage genetic optimization procedure. Its main ideas are 2-fold: (1) it encodes all the channels of the pertained network and divide them into multiple searching spaces according to the different functional convolutional layers from concrete to abstract. (2) it develops a wavelet channel aggregation based fitness function to explore the most representative and discriminative channels at each layer and prune the network dynamically. In the experiments, the proposed GWCS is evaluated on CIFAR-10, CIFAR-100, and ImageNet datasets with two kinds of popular deep convolutional neural networks (CNNs) (ResNet and VGGNet). The results demonstrate that GNAS outperforms state-of-the-art pruning algorithms in both accuracy and compression rate. Notably, GNAS reduces more than 73.1% FLOPs by pruning ResNet-32 with even 0.79% accuracy improvement on CIFAR-100.

## 1. Introduction

Deep convolutional neural networks (CNNs) have achieved substantial progress in many research fields, such as computer vision (Wang et al., 2019), natural language processing (Giménez et al., 2020), and information recommendation (Wu et al., 2021a,b). However, the number of parameters in deep CNN-based models (e.g., ResNet-50 He et al., 2016) generally exceeds hundreds of megabytes. It needs billions of floating number operations (FLOPs) to run these deep models, bringing a significant challenge to deploy large networks on devices with limited resources (e.g., mobile phone, robot, drone). Thus, the huge storage and the expensive computational costs have become significant problems to hinder practical applications of deep CNNs in complex real-world scenarios.

Neural network compression (Renda et al., 2020; Xu et al., 2020) has been proposed to accelerate the deep CNNs computation. Network pruning is one of the most intuitive methods to create a small-scale network by reducing redundant and non-informative weights (Li et al., 2016; Yang et al., 2017). The critical point in network pruning is finding a proper metric to measure the importance of the pruned parts. One solution is deleting the weights with small absolute values (Liu et al., 2017) under the presumption that the smaller value of a weight parameter is, the less impact it has on the final result. But this intuitive assumption has been proved invalid in some cases (Ye et al., 2018). On the other hand, many other pruning algorithms have been developed, such as judging the influence of parameter clipping on training loss (Molchanov et al., 2016) or the reconstruction errors of feature outputs (He et al., 2017). However, such algorithms mainly rely on human expert knowledge and hand-crafted pruning rules.

In addition, prevailing methods usually ignore the variety of channels among layers (He et al., 2018, 2019). The candidates of sub-networks are chosen according to various evaluation criteria with the pre-defined pruning ratio (PR) for each layer or block. In this case, no matter which specific channels are pruned, the compressed network architecture remains the same. As mentioned in Gu et al. (2018) and He et al. (2020), the channels of different layers have various functions. Thus, the truly informative (or discriminative) channels might be wrongly removed if the PR is fixed (Yang et al., 2018; Liu et al., 2019), resulting in a decrease in the test accuracy of the pruned network. Furthermore, these manually-set pruning parameters may be the sub-optimal trade-off between the model size and prune accuracy.

Recently, automatic pruning algorithms with neural architecture search (NAS) approaches (Chen et al., 2021; Jia et al., 2021; Liang et al., 2021; Wang et al., 2021a; Xu et al., 2021; Yang et al., 2021) are identified as a promising way to automate network compression. It casts the network pruning problem into the NAS framework, i.e., the search space of NAS is the parameters of the pre-trained network to be pruned. A typical NAS-based pruning model (Dong and Yang, 2019; Jiahui and S., 2019; Liu et al., 2019) explores the potential sub-network architectures from the pre-trained network. Then the intermediate compressed model is evaluated and fine-tuned sequentially to construct the final output. However, prevailing NAS-based algorithms (Jiahui and S., 2019; Liu et al., 2019) usually simplify the network at a coarse-grained level while ignoring the critical specific channels.

**Figure 1:**
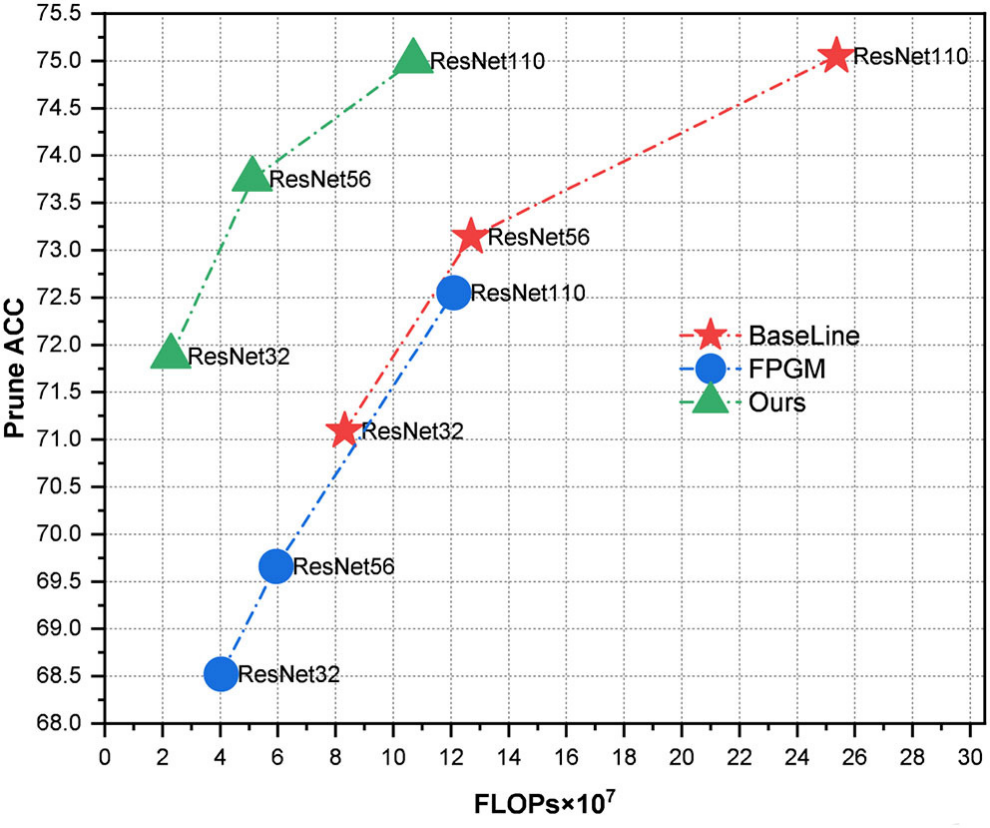
We compare classification accuracy vs. computational complexity (FLOPs) with ResNet series models on CIFIA-100. Our pruning method with a more flexible optimization procedure obtains more promising results than filter pruning algorithm based on geometric median (FPGM) (He et al., 2019) with the fixed pruning rate.

This study proposes a novel NAS-based pruning model named GWCS. It can dynamically prune a pre-trained network at the channel level while maintaining the model accuracy. First, we formulate the network compression task as a combinatorial optimization problem. Specifically, we genetically encode each channel in the pre-trained network and prune it adaptively using a dynamic selection operation in multiple stages with a wavelet channel aggregation (WCA) based fitness function. As shown in Figure 1, our dynamic network pruning model yields much higher prune accuracy than the hand-crafted pruning method for ResNet series models on CIFIA-100. Notably, our model even accelerates ResNet32 and ResNet56 three times, along with the improved classification results.

This study makes innovative contributions in the automatic network pruning process for image classification as follows:

(1) We develop a GWCS pipeline to prune the pre-trained network dynamically. It models the channel-wise network pruning task as a multi-stage genetic optimization procedure. (2) We introduce a WCA based fitness function to evaluate and exploit the most informative channels. (3) Extensive experiments are conducted to demonstrate the effectiveness of the proposed dynamic channel pruning model on some popular benchmark datasets, including CIFAR-10, CIFAR-100, and ImageNet. Our GWCS outperforms the tested state-of-the-art models regarding pruning accuracy and network compression rate.

The rest of the study is organized as follows: Section 2 presents the proposed genetic wavelet channel search scheme. The experimental results are provided in section 3, following the discussion in section 4.

## 2. Methods

### 2.1. Overview of GWCS

**Figure 2:**
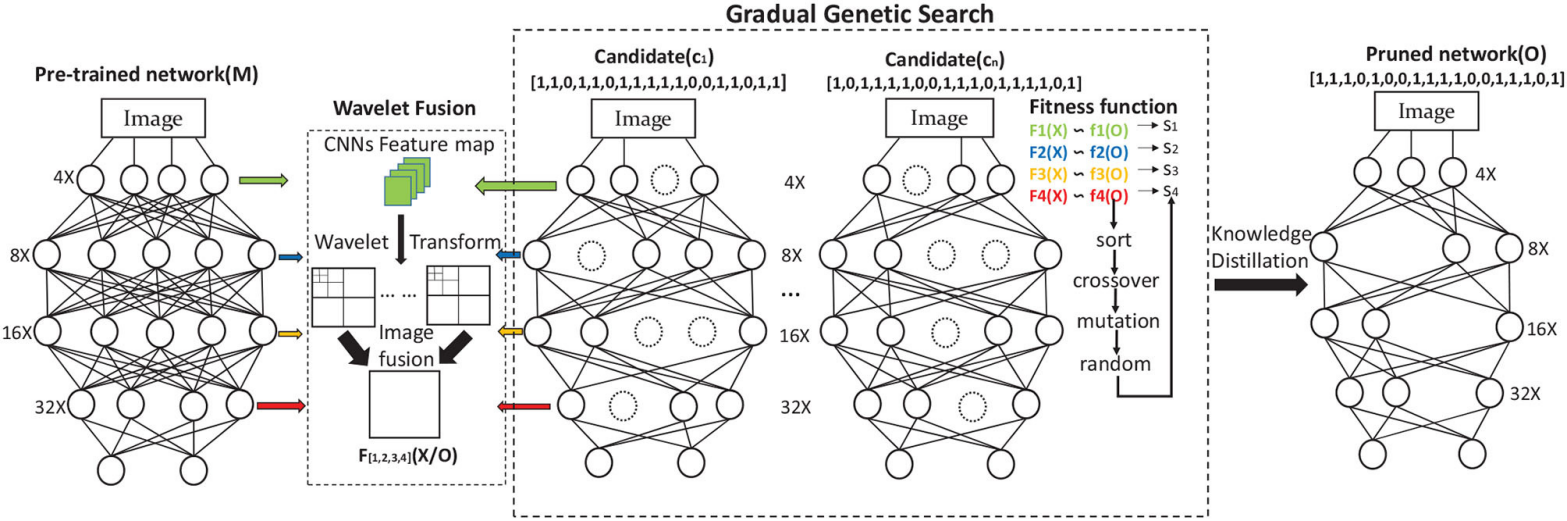
Overview of GWCS model. The input CNNs *M* is the pre-trained model. The circles in figures mean channels. The pre-trained CNNs *M* can be encoded and pruned through GGS during the iteration process. Finally, the pruned network containing the most informative channels can be fine-tuned by KD.

This study aims to remove the redundant channels from the pre-trained network *M* for generating a pruned output *O* with reliable classification results. We approach the problem of compressing the network with flexible pruning layers as a genetic search framework. It contains three steps which are shown in Figure 2: (1) Training a large CNNs (the pre-trained network *M*), (2) Using GWCS to prune the channels in pre-trained network *M* layer by layer, (3) Knowledge distilling (KD) the pruned network to recover the model accuracy. In the search process, the most critical part is to effectively and flexibly remove the inadequate channels in the pre-trained network *M* without significantly compromising accuracy. Next, we will introduce our GWCS model to address this problem.

### 2.2. Genetic Wavelet Channel Search

#### 2.2.1. Gradual Genetic Search (GGS)

**Initialization**. Our proposed GWCS strategy is an iterative process in which the initial network is made gradually better as a group called a population. At first, all the channels of pre-trained network *M* can be encoded into random binary genotypes to generate the population A, in which we denote the candidate compressed network ${\text{X}}_{i}\in A$ standing for the *i*th instance in A:


(1) $${\text{X}}_{i}=\left\{c_{i}^{1},c_{i}^{2},\ldots ,c_{i}^{N}\right\}$$

where *i* ∈ {1, 2, …, *NP*}, and *NP* and *N* is the total number of population individuals. *N* is the total number of the channels in **X**_*i*_, and $c_{i}^{j}$ means the *j*-th channel code of **X**_*i*_, while $c_{i}^{j}=0$ represents the corresponding channel to be pruned; otherwise, $c_{i}^{j}=1$ means the channel will be reserved.

All the individuals of **X**_*i*_ are grouped into the population set A, defined in Equation (2):


(2) $$A=\{\begin{array}{l} X_{1}=\left[1,0,1,1,0,1,0,\ldots ,0,1,1,1\right]\\ X_{2}=\left[0,1,1,0,0,1,0,\ldots ,0,1,0,1\right]\\ \vdots \\ \underset{channels^{\prime }\;code}{\underset{︸}{X_{NP}=\left[1,0,1,0,0,1,1,\ldots ,0,0,1,0\right]}} \end{array}$$

**Gradual Genetic Search**. Searching the entire space with millions of channels in **X**_*i*_ is intractable. In this study, we proposed a new strategy, named GGS, to examine the valuable channels hierarchically, rather than directly inspecting all the $c_{i}^{j}$ in **X**_*i*_ as a whole.

The success of CNN mainly attributes to its hierarchical structures from the concrete level to the abstract level, i.e., the convolutions in shallow layers extract coarse features such as color and edges. In contrast, those in deep layers acquire more abstract or semantic features related to the concept of category. The proposed GGS is consistent with this theory. As shown in Figure 2, we divide the neural network searching process into multiple stages according to the down-sampling sizes in CNNs, i.e., we can divide the whole search space into several sub-spaces with multi-scale feature sizes down-sampling from 4× to 32×, e.g., an individual network **X**_*i*_ can also be divided as:


(3) $${\text{X}}_{i}=\left[{\text{X}}_{i}^{\left(1\right)},{\text{X}}_{i}^{\left(2\right)},{\text{X}}_{i}^{\left(3\right)},{\text{X}}_{i}^{\left(4\right)}\right]$$

where the sub-network ${\text{X}}_{i}^{\left(st\right)}\in {\text{X}}_{i}$ and *st* ∈ [1, 4]. Note that the maximum iteration number of *T*^(*st*)^ is set variously in each stage due to the total number of channels in ${\text{X}}_{i}^{\left(st\right)}$ is different.

**Crossover**. In every iteration, we can produce a new group of offspring (i.e., new codes of the pruned network) using variations through the crossover operator. First, we randomly selected two chromosomes as parents, e.g., ${\text{X}}_{r1}^{\left(st\right)}$ and ${\text{X}}_{r2}^{\left(st\right)}$ are chosen to exchange channel bits at certain points. After that, a new offspring ${\text{X}}_{cr}^{\left(st\right)}$ can be generated by using the multipoint crossing strategy based on the selected parents ${\text{X}}_{r1}^{\left(st\right)}$ and ${\text{X}}_{r2}^{\left(st\right)}$, which can be formulated as:


(4) $${\text{X}}_{cr}^{\left(st\right)}=\text{G}◦\left({\text{X}}_{r1}^{\left(st\right)}\right)+|1-\text{G}|◦\left({\text{X}}_{r2}^{\left(st\right)}\right)$$

where **G** is a random vector of bits (0 or 1) to disrupt the codes of selected parents.

**Mutation**. The mutation operator is applied to further enhance the diversity of offspring and the ability of the model to escape from local optimization. We use the binary mutation strategy by flipping the bit randomly in ${\text{X}}_{cr}^{\left(st\right)}$ to produce a new individual ${\text{X}}_{m}^{\left(st\right)}$, defined as follows.


(5) $${\text{X}}_{m}^{\left(st\right)}=H\left({\text{X}}_{cr}^{\left(st\right)}\right)$$

where *H*(·) means that a total of *p*_*m%* of binary codes in randomly selected channels will be flipped. In our study, the bit flip in the genotype space could potentially create a different pruned network.

**Selection**. Every candidate of the lightweight network in the population (including both parents and offspring) will be evaluated for survival and reproduction (becoming a parent) in each iteration. For each network at stage ${\text{X}}_{i}^{\left(st\right)}$, the top K individuals with the highest fitness are selected based on the Roulette Wheel algorithm with a survival probability of *p*_*s%*, to form the next generation. In this study, the $P_{i}^{\left(st\right)}$ of each ${\text{X}}_{i}^{\left(st\right)}$ at the *st*_*th*_ stage can be denoted as follows:


(6) $$P_{i}^{\left(st\right)}=\frac{Ft\left({\text{X}}_{i}^{\left(st\right)}\right)}{\sum_{i=1}^{NP}Ft\left({\text{X}}_{i}^{\left(st\right)}\right)}$$

where the *Ft*(·) is the fitness function, which determines whether a potential pruned network could survive and will be introduced in detail below.

#### 2.2.2. Fitness Function

In GWCS, we aim to find the best individual network after removing the redundancy channels through the fitness evaluation. Considering that the most informative channels should have the minimal reconstruction error of feature maps, our fitness function *Ft*(·) is designed based on the similarity of feature maps between the pre-trained network and pruned network. The output *Ft* can be used as a pruning criterion to identify the best-pruned networks.

Wavelet transform has been successfully applied in image processing. Its primary purpose is to extract the specific properties of the image with the wavelet basis function, which can be formulated as:


(7) $$F^{⋆}=\frac{1}{\sqrt{a}}\int_{-\infty }^{+\infty }F*\psi \left(\frac{t-\tau }{a}\right)dt$$

where *a* is the scale that controls the stretching of the wavelet, and **τ** is the translation that affects the translation of the wavelet. *F* represents the feature maps of the last CNN layer in the input network. In our GWCS algorithm, we adapt the Haar wavelet function (Porwik and Lisowska, 2005) to extract the frequency features due to its simplicity and effectiveness.

To calculate the similarity between the networks with different sizes of features maps (i.e., the total number of channels of the network is variable after the dynamic pruning), we aggregate all the wavelet feature maps into one vector, which is formulated in Equation (8).


(8) $$F^{*}=\max\left(F_{HH}^{⋆}\right)⊕Avg\left(F_{LL}^{⋆}\right)$$

where *HH* and *LL* represent high-frequency and low-frequency information. ⊕ is the element-wise addition. The final fused feature vectors F^*^ are generated by the maximum values of *HH* and the average values of *LL* using ⊕ operation. Comparing to conventional aggregate functions, including global average pooling (GAP) or global max pooling (GMP), more rich information contained in both high- and low-frequency components are more helpful for improving the classification (Qin et al., 2020), i.e., it can further boost the feature similarity estimation.

**Figure 3:**
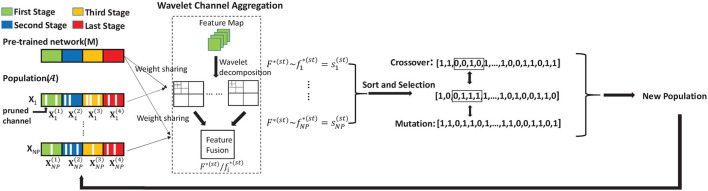
Details of GGS. We divide the network searching process into multiple stages with different maximum iterations numbers. The final output is the code of the pruned network.

As is illustrated in Figure 3, both *F*^(*st*)^ and $f_{i}^{\left(st\right)}$ can be transformed and aggregated by wavelet operation using (Equations 7, 8), denoted as F^*(*st*)^ and $f_{i}^{*\left(st\right)}$, respectively. We can obtain the similarity $s_{i}^{\left(st\right)}$ based on the cosine distance, which can be formulated as:


(9) $$s_{i}^{\left(st\right)}=\frac{F^{*\left(st\right)}\cdot f_{i}^{*\left(st\right)}}{∥F^{*\left(st\right)}∥∥f_{i}^{*\left(st\right)}∥}$$

The best-pruned network *O* can be achieved by selecting the best individual with the highest fitness from population A after maximum iterations. The detailed steps of GGS are shown in Algorithm 1, from which we can observe that the time complexity of our GWCS is *O*[*st* * *T*^*st*^ * (*NP* * *N* * *size*(*F*) + *NP* * *N* + *N*)].

**Algorithm 1:** Algorithm of the gradual genetic search.

**Input**: The original network *M*	
**Output**: A pruned network *O*	
1:	Randomly initialize the binary codes in networks {**X**_1_, **X**_2_, ⋯ , **X**_*NP*_} to form the initial population $A_{0}$ by Equation (2).
2:	Set the maximum iteration number **T**=[*T*^1^,…,*T*^4^] for each searching stage.
3:	**for** *st = 1 to 4* **do**
4:	**while** *t* in *T*^*st*^ ***do***
5:	*Calculate* F^*(*st*)^ *and* ${\left\{f_{i}^{*\left(st\right)}\right\}}_{i=1}^{NP}$ *by Equation(7, 8)*.
6:	*Calculate the fitness* ${\left\{s_{i}^{\left(st\right)}\right\}}_{i=1}^{NP}$ *by Equation (9)*.
7:	*Select top K individuals from* $A_{t}^{\left(st\right)}$ *by Eq. (6)*.
8:	*Crossover and mutate the top K individuals using (Equation 4, 5)*.
9:	*Generate*$A_{t+1}^{\left(st\right)}$
10:	*Update* *t* = *t* + 1
11:	***end*** ***while***
12:	**end for**
13:	Select the best individual as the pruned network *O*
14:	**return** *O*

### 2.3. Knowledge Distillation

The fine-tuning (FT) process is crucial for recovering the original performance (Dong and Yang, 2019). In this study, knowledge distillation (KD) (Hinton et al., 2015) is applied to improve the performance of the pruned network. In our model, the pruned network derives from the pre-trained network. Thus, we take the pre-trained network as the teacher network and transfer its knowledge into the pruned network (i.e., the student network).

In the classification task with CNNs, the softmax layer is adopted as the classifier. The softmax output is a one-hot vector, i.e., the classification result is the label with the largest value. However, such logit outputs contain very little information as we cannot learn the relationship between classes except the prediction labels. The output results can be further softened as:


(10) $$q_{k}=\frac{exp\left(z_{k}/T\right)}{\Sigma_{j}exp\left(z_{j}/T\right)}$$

where *z* is the softmax vector from the pre-trained network. *T* stands for temperature. When *T* tends to zero, the output *q*_*k*_ is degraded into the one-hot vector. The pruned network can take the soft target output *q*_*k*_ as the training loss to transfer the knowledge from the original unpruned network.

Following the prevailing study in Dong and Yang (2019), we use the middle layer transfer of KD to optimize the searched network *via* (Equation 11).


(11) $$L=\rho_{1}L_{1}+\ldots +\rho_{n}L_{n}+\left(1-\rho_{1}-\ldots -\rho_{n}\right)L_{hard}$$

where *L* is the total loss function of KD and *L*_*n*_ is the loss function of each training stage.

## 3. Results

### 3.1. Experimental Setting

#### 3.1.1. Datasets

In our experiment, we evaluated the tested models on CIFAR-10, CIFAR-100 (Krizhevsky, 2009), and ImageNet (Russakovsky et al., 2015) for image classification tasks. CIFAR-10 consists of 50 k training and 10 k testing 32 × 32 images in 10 classes. Similar to CIFAR-10, the CIFAR-100 dataset has 100 categories. There are 500 training images and 100 verification images for each class. The ImageNet dataset (ISLVRC 2012) (Russakovsky et al., 2015) is a large visual database collected from the real world. it consists of 1,281,167 training images and 50,000 validation images in 1,000 classes. Data augmentation techniques, including random resize, crop, brightness changing, and horizontal flipping are also employed to improve accuracy.

#### 3.1.2. Implementation Details

Following the previous studies (He et al., 2018, 2019; Dong and Yang, 2019), ResNet series networks (He et al., 2016), and VGGNet-16 (Simonyan and Zisserman, 2015) are chosen as the baseline networks in our pruning experiment. We trained them using the standard stochastic gradient descent (SGD) optimization with batch size 128. Our initial learning rate is set to 0.1, which is gradually reduced with a weight decay of 0.0005. For CIFAR-10, we train the Resnet for 150 epochs and train the VGGNet-16 for 200 epochs, respectively. For CIFAR-100, we train the Resnet for 200 epochs and train the VGGNet-16 for 300 epochs, respectively. For ImageNet, we train the Resnet for 150 epochs and train the VGGNet-16 for 300 epochs, respectively. All models are implemented on dual NVIDIA GTX1080ti GPUs in PyTorch.

#### 3.1.3. Specific Searching and Training Setting

We take the unpruned network as the initial input for our algorithm in the process of pruning. First, the codes of 50 individuals (which have the same number of channels as the unpruned network) are randomly initialized. Each individual will be evaluated as a candidate pruned network. Then, we search the optimal channels using GGS in multiple stages, i.e., we divided the searching procedure into four stages with the maximum number of iterations in [10, 10, 5, 5]. The top 20 individuals are chosen for crossover and mutating based on the fitness values. Specifically, in crossover operation, two individuals are randomly selected for exchanging 50% of codes with each other. In mutation, 10% of codes of individuals are chosen for mutating, i.e., 0 and 1 interchange. Finally, a new population can be generated by selecting the top 30 individuals for the next iteration.

### 3.2. Comparison With State-of-the-Art Methods

We compare several state-of-the-art network pruning models published in most recent years in our experiments.

**Soft Filter Pruning (SFP):** He et al. (2018) proposes a SFP method. After training the model at each epoch, the L2 norm of the corresponding channel is calculated. Meanwhile, the lower-ranked channel is set to zero according to a manual pruning rate. Still, the pruned ones will also participate in the next round of iterations instead of deleting them directly.

**Discrimination aware channel pruning (DCP):** Zhuang et al. (2018) implements a pruning method called DCP, which adds discriminative losses into the network and obtains pruned network after a greedy algorithm for channel selection.

**Genetic channel pruning (GCP):** Hu et al. (2018) also uses a genetic algorithm to code and prune the network. However, the GCP searches the entire pre-trained network as a whole and prunes it with a group of manually assigned compression rates and the layer-wise error is estimated with the Hessian matrix.

**Filter pruning algorithm based on geometric median (FPGM):** He et al. (2019) proposes a filter pruning algorithm based on geometric median. FPGM deletes the redundant filters instead of the relatively less important ones with a manual setting of pruning rate.

**Transformable architecture search (TAS):** Dong and Yang (2019) proposes a TAS approach for compressing CNNs by channel-wise probability distribution and knowledge transfer. TAS aimed to search for the appropriate width and depth of the pruned network.

**High-rank pruning (HRank):** Lin et al. (2020) reveals a rule of CNNs even if the input image is different, there is always a large rank in the same part of the feature graph. The results suggest that the latent rank information is essential in the network so that the redundancy weights can be compressed with low-rank feature maps.

**Joint search-and-training (JST):** Lu et al. (2020) implements an automatic search algorithm by training and pruning simultaneously. It saves the pre-training time in the automatic pruning algorithm with competitive classification accuracy.

**Discrete model compression (DMC):** Gao et al. (2020) proposes a discrete compression model, which attaches a gate for each channel to control whether the channel is opened or not. Then the pruned network is obtained by gradient descent to optimize the gate parameters.

**Learning filter pruning criteria (LFPC):** He et al. (2020) introduces a LFPC to select a set of suitable measures for different layers adaptively. LFPC evaluates the importance of the filters based on the proposed differentiable criteria sampler with Gumbel-softmax.

**Structural redundancy reduction with graph redundancy (SRR-GR):** Wang et al. (2021b) assumes that the performance of the pruning filter in the more redundant layer is better than that of pruning the least important filter in all layers. Based on this assumption, this method establishes an undirected graph for each layer, in which each vertex represents a filter and edge denotes the distance between filter weights. The quotient space size and covering number are calculated according to the redundancy rates of each graph.

**Manifold regularized dynamic pruning (ManiDP):** Tang et al. (2021) develops a (ManiDP) strategy that identifies the complexity and feature similarity of the training data set. The network is pruned dynamically by exploiting the manifold regularization, and the appropriate sub-network is allocated for each instance.

### 3.3. Main Results With ResNet

#### 3.3.1. Results on CIFAR-10 and CIFAR-100

The pruning result of ResNet series networks on CIFAR-10 and CIFAR-100 are shown in Tables 1, 2. Among all the tested pruning algorithms, our GWCS model consistently reduces the largest number of channels to generate the minimum FLOPs among all the tested pruning models. Notably, our model produced the highest pruning rates of 73.6 and 73.1% by pruning ResNet-32 on CIFAR-10 and CIFAR-100, respectively. It saved more than half of GPU computational cost compared to FPGM. Turning to the pruning accuracy, our model achieves the lowest accuracy drops by pruning the ResNet networks on CIFIA-10 and obtains the best prune accuracy with ResNet-56 and ResNet-110 on CIFIA-100. For example, when pruning ResNet-110, our model achieves the highest pruning accuracy of 75%, outperforming the second-best model (FPGM) by more than 1.54% in terms of accuracy drop, along with much fewer computations. Note that the proposed GWCS model also achieves a very close result (only 0.05% of the drop of accuracy) to the original ResNet-110 with the highest FLOPs reduction (nearly 2.39 × compression rate). These results suggest that the proposed GWCS is an effective and reliable network pruning model, achieving a better trade-off between pruning accuracy and model size.

**Table 1:** Comparison results on CIFAR-10 with ResNet-32, 56, and 110.

**Network**	**Method**	**Baseline Acc (%)**	**Prune Acc (%)**	**Drop (%)**	**FLOPs(PR)**
	FPGM	92.63	92.31	0.32%	4.03E7(41.5%)
	SFP	92.63	92.08	0.55	4.03E7(41.5%)
ResNet-32	TAS	93.88	92.92	0.96	3.78E7(45.4%)
	LFPC	92.63	92.12	0.51	3.27E7(52.6%)
	ManiDP	92.66	92.15	0.51	2.54E7(63.2%)
	Ours	93.08	92.97	**0.11**	**1.82E7(73.6%)**
	HRank	94.46	93.52	0.94	6.58E7(37.9%)
	JST	94.41	93.68	0.73	6.32E7(49.7%)
ResNet-56	FPGM	93.59	92.89	0.70	5.94E7(52.6%)
	SFP	93.59	92.26	1.33	5.94E7(52.6%)
	TAS	94.46	93.69	0.77	5.95E7(52.7%)
	Ours	94.23	93.75	**0.48**	**5.05E7(60.3%)**
	SFP	93.67	92.97	0.70	1.21E8(52.3%)
ResNet-110	TAS	94.97	94.33	0.64	1.19E8(53.0%)
	LFPC	93.68	93.07	0.61	1.01E8(60.3%)
	Ours	95.03	94.78	**0.25**	**1.12E8(56.0%)**

*The best results are highlighted in bold and the second-best results are underlined. “Drop” means accuracy drop, “FLOPs (PR)” represents FLOPs of the compressed model with the corresponding pruning ratio (PR)*.

**Table 2:** Comparison results on CIFAR-100 with ResNet-32, 56, and 110.

**Network**	**Method**	**Baseline Acc (%)**	**Prune Acc (%)**	**Drop (%)**	**FLOPs(PR)**
	FPGM	69.77	68.52	1.25	4.03E7(41.5%)
ResNet-32	TAS	70.62	71.74	**–1.12**	3.80E7(45.0%)
	Ours	71.09	71.88	–0.79	**2.29E7(73.1%)**
	FPGM	71.41	69.66	1.75	5.94E7(52.6%)
ResNet-56	JST	72.89	70.63	2.26	6.72E7(51.1%)
	TAS	73.18	72.25	0.93	6.12E7(51.3%)
	Ours	73.14	73.75	**–0.61**	**5.12E7(59.7%)**
	FPGM	74.14	72.55	1.59	1.21E8(52.3%)
ResNet-110	JST	74.42	72.26	2.16	1.08E8(58.0%)
	TAS	75.06	73.16	1.90	1.20E8(52.6%)
	Ours	75.05	75.00	**0.05**	**1.07E8(58.2%)**

*The best results are highlighted in bold and the second-best results are underlined*.

#### 3.3.2. Results on ImageNet

The effectiveness of GWCS is further validated by the transferred performance on ImageNet using ResNet-50 and ResNet-101. As shown in Table 3, The proposed approach can produce a promising test accuracy (0.36 and 0.33% Top-5 accuracy drop on ResNet-50 and ResNet-101, respectively) with the largest compression rates. For example, GWCS outperforms TAS by 0.12% Top-5 accuracy drop with a significant FLOPs reduction (less than nearly 15.6%). When pruning RedNet-101, our model obtains a comparable test accuracy rate but removes nearly 2 × FLOPs than the SFP model.

**Table 3:** Comparison results on ImageNet with ResNet-50 and ResNet-101.

**Network**	**Method**	**Top-1** **Prune Acc (%)**	**Top-5** **Prune Acc (%)**	**Top-1** **Drop (%)**	**Top-5** **Drop (%)**	**FLOPs(PR)**
	HRank	74.98	92.33	2.48	1.22	2.62E9(40.8%)
	TAS	76.20	93.07	1.26	0.48	2.31E9(43.5%)
	JST	75.51	92.43	1.01	0.66	2.25E9(44.9%)
	FPGM	74.83	92.32	1.32	0.55	2.58E9(53.5%)
ResNet-50	DMC	75.35	92.49	**0.80**	0.38	2.01E9(55.0%)
	SRR-GR	75.76	92.67	1.02	0.51	2.01E9(55.1%)
	DCP	74.95	92.32	1.06	0.61	1.99E9(55.6%)
	Our	76.64	93.78	1.09	**0.36**	**1.83E9(59.1%)**
	SFP	77.51	93.71	**–0.14**	**–0.20**	6.43E9(30.0%)
ResNet-101	FPGM	77.37	93.56	0.05	0.00	6.43E9(30.0%)
	Our	77.65	93.65	–0.13	0.33	**4.36E9(58.7%)**

*The best results are highlighted in bold, and the second-best results are underlined*.

**Figure 4:**
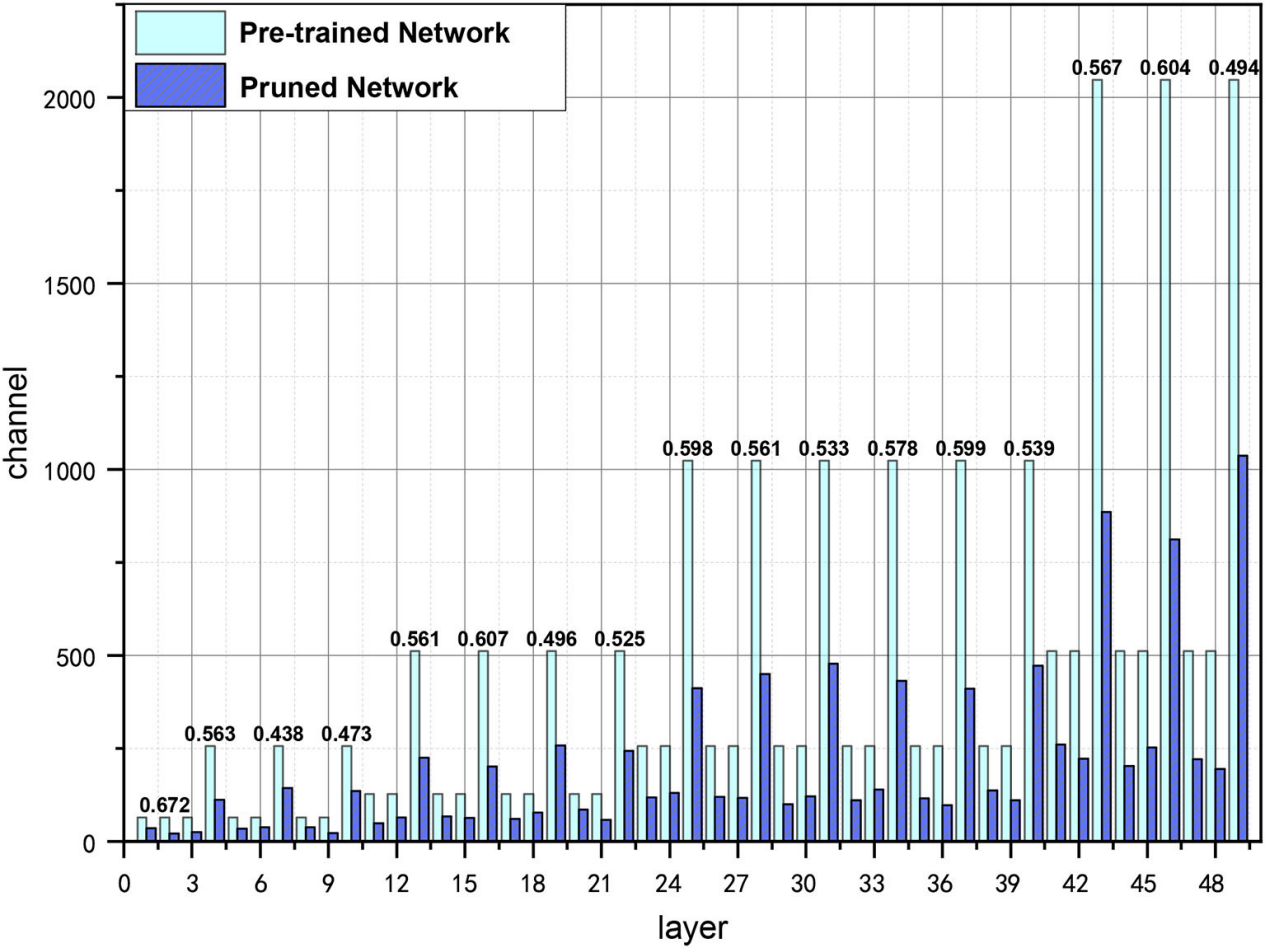
Visualization of pruned channels in ResNet-50 with GWCS on ImageNet, where layer on the x-axis represents the number of layers in ResNet-50, channel means the number of channels in each layer. The bars in light blue indicate the number of channels in the pre-trained network. The purple ones indicate the number of channels after pruning. The PR are shown on the top of the bars.

We also visualize the pruned channels of ResNet-50 on ImageNet in Figure 4. As can be seen from Figure 4, the pruning rates are various in each layer, which could be more suitable for channel searching as the information contained in layers may be different, and the truly useful channels can be preserved with a flexible pruning strategy.

### 3.4. Main Results With VGGNet

In Table 4, we show the comparison results in terms of Prune ACC and FLOPs on CIFAR-10 and CIFAR-100 with VGGNet-16. Among all the tested models, our proposed GWCS still achieves the highest pruning rate and yields the lowest FLOPs. In particular, we can see that the pruning reductions of our model are 64.18 and 66.61% on CIFAR-10 and CIFAR-100, respectively, which are much higher than HRank, GCP, and JST. Furthermore, our model produces a comparable accuracy with much fewer FLOPs. For instance, compared with GCP, our model obtains a very close test accuracy (0.58 vs. 0.20% accuracy drop) but prunes more than 1.8 × channels on CIFAR-100.

**Table 4:** Comparing our model and other methods with VGGNet-16 on CIFAR-10 and CIFAR-100.

**Datasets**	**Method**	**Baseline Acc (%)**	**Prune Acc (%)**	**Drop (%)**	**FLOPs(PR)**
	GCP	92.71	92.74	**–0.03**	2.74E8(52.0%)
CIFAR-10	HRank	93.96	93.43	0.53	2.71E8(53.5%)
	Ours	94.13	93.76	0.37	**2.29E8(64.18%)**
fine-tuning	GCP	72.21	72.01	**0.20**	3.82E8(37.0%)
CIFAR-100	JST	75.75	74.63	1.12	3.22E8(45.0%)
	Ours	73.75	73.17	0.58	**2.21E8(66.61%)**

*We highlight the best and second-best results in bold face and underline, respectively. “Drop” means accuracy drop, “FLOPs (PR)” means FLOPs and pruning rate*.

## 4. Discussion

### 4.1. GGS vs. Overall Genetic Search

In our model, we proposed a hierarchical search method named GGS algorithm to prune the network in multiple stages instead of searching the whole space of all channels [i.e., Overall Genetic Search (OGS)] at each iteration. We conduct the ablation experiment for studying the effect of GGS comparing to the overall search method on the CIFAR-10 dataset using ResNet-32. The channels of the pre-trained network are divided into four stages by using GGS, and the maximal number of iterations in each stage is set to 10, 10, 5, and 5, respectively. Thus, the total number of iterations in the pruning process is 30, which is also set as the maximum iteration number for OGS. The same FT operation with KD is applied in the OGS method to recover the accuracy of the pruned network. We can inform from Table 5 that, GGS generates a more accurate classification result with more than 0.9% FLOPs reduction, compared to the OGS method when pruning ResNet-32 on CIFAR-10, suggesting that the proposed GGS proves a more optimal solution for identifying the critical channels in a large search space.

**Table 5:** Comparison of gradual search and overall search on CIFAR-10 with ResNet-32.

**Method**	**Prune Acc(Drop)**	**FLOPs(PR)**
Overall Genetic Search	92.19%(0.89%)	2.31E7(72.7%)
Gradual Genetic Search	**92.97%(0.11%)**	**2.23E7(73.6%)**

*The best results are highlighted in bold, and the second-best results are underline*.

### 4.2. Effect of WCA

Wavelet channel aggregation in the proposed GWCS model is used to evaluate the performance of the pruned network based on the fused wavelet transformed features. Comparing to conventional feature aggregation methods used in deep CNNs, including GAP, GMP, and GAP+GMP, we investigated the utility of the fitness function based on the WCA method on CIFAR-10 and CIFAR-100 in terms of prune accuracy and FLOPs. The comparison results are reported in Table 6. We observed that GAP prunes much more channels while producing much worse accuracy values. However, WCA achieves the best classification accuracy with the comparable FLOPs. As mentioned in Qin et al. (2020), GAP extracts the low-frequency information (e.g., the contour of an object) of the image, while GMP takes the high-frequency information (e.g., edge or texture). Nevertheless, both the contour and texture features are essential for image classification. Therefore, considering both high- and low-frequency information in our WCA method could help identify the best-compressed network.

**Table 6:** Comparison of different feature aggregation methods applied in fitness functions on CIFAR-10 with ResNet-32.

**Fitness function**	**Prune Acc(Drop)**	**FLOPs(PR)**
GAP	91.41%(1.67%)	**2.15E7(75.4%)**
GMP	91.25%(1.83%)	2.21E7(74.0%)
GAP+GMP	91.84%(1.24%)	2.23E7(73.5%)
WCA	**92.97%(0.11%)**	2.23E7(73.6%)

*The best results are highlighted in bold, and the second-best results are underlined. GAP is global average pooling. GMP is global max pooling. WCA is our proposed wavelet channel aggregation method*.

**Figure 5:**
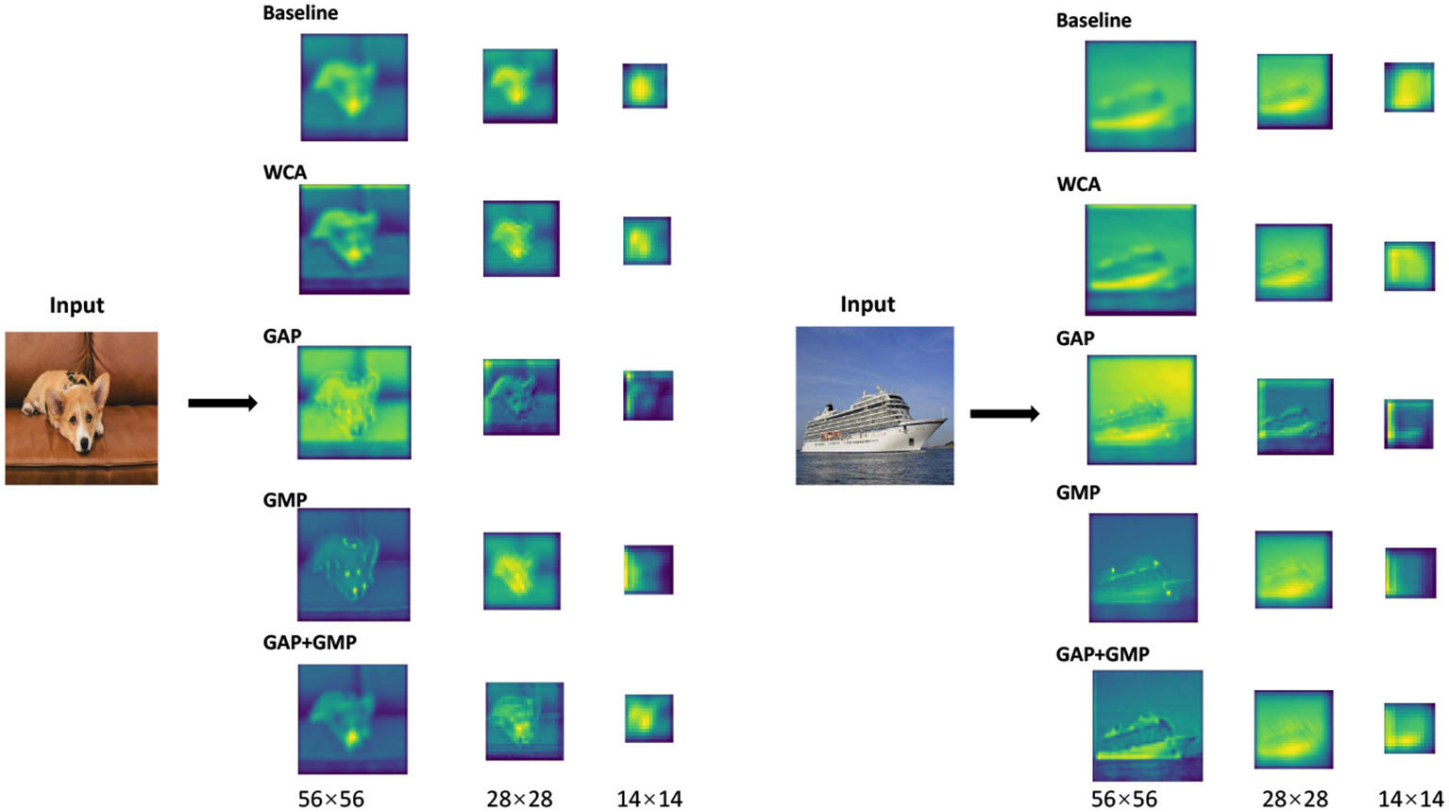
Visualization of the feature maps of the pruned network obtained with different feature aggregation functions. Baseline is the pre-trained ResNet-50, GAP is the global average pooling, GMP is the global max pooling, and WCA is our wavelet channel aggregation (WCA).

As is shown in Figure 5, the feature maps of the pruned network with selected channels based on the WCA contains more information for categorization than those of the selected ones using other conventional feature aggregation methods. The results further prove that the proposed WCA can adequately choose the channels with the most representational power for the network.

### 4.3. Effect of FT Strategies

Knowledge Distillation (KD) is the last step in our GWCS model for regaining the lost performance. In this sub-section, we try to investigate the effectiveness of the GWCS for reducing the redundancy channels instead of relying on KD technology alone. Thus, two ablation experiments, i.e., the GWCS based on KD (GWCS+KD), are conducted to compare with: (1) the conventional FT technique by retraining the compact network from scratch, named GWCS+FT. (2) The channels pruning strategy with random selection and KD, named RS+KD.

**Figure 6:**
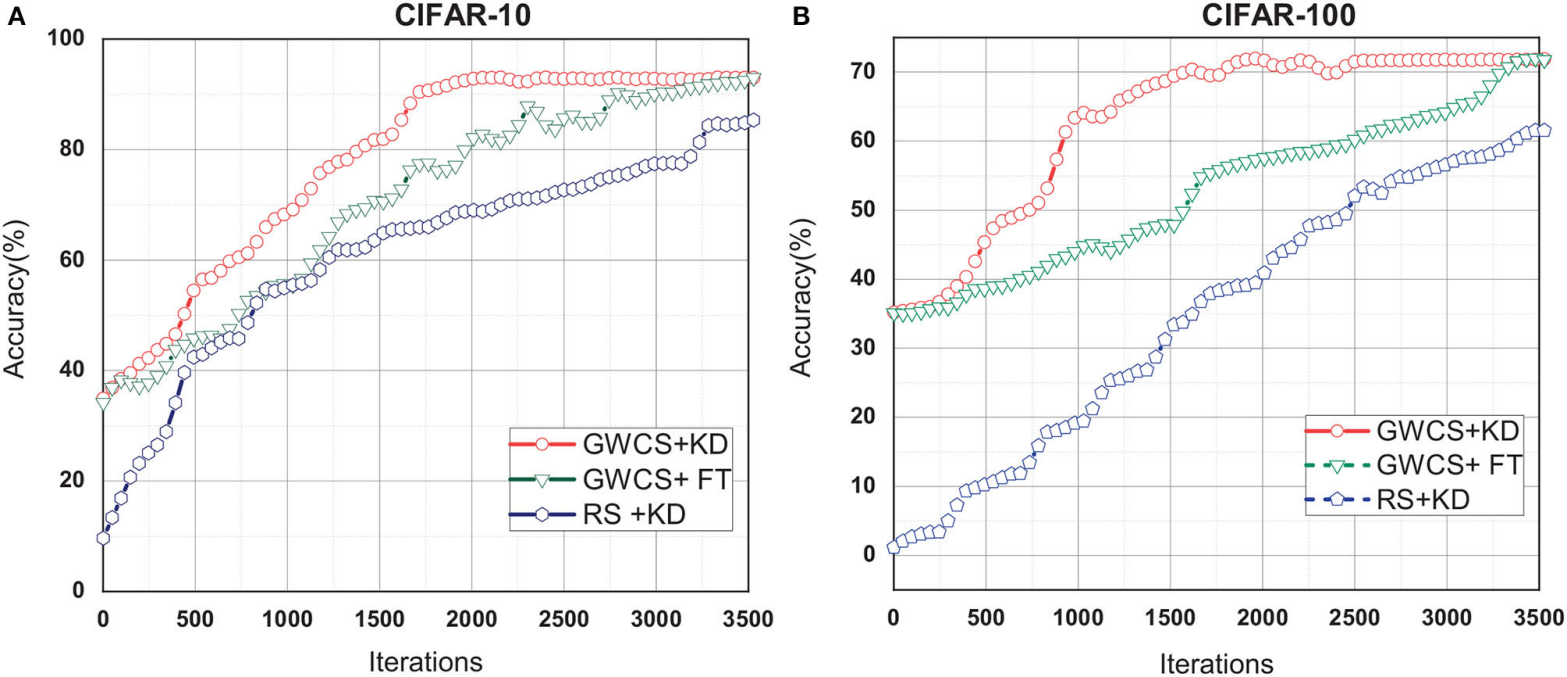
Test the effect of fine-tuning (FT) strategies on CIFAR-10 and CIFAR-10 with ResNet-32. **(A)** FT results on CIFAR-10. **(B)** FT results on CIFAR-100.

As shown in Figure 6, we can observe that KD and FT result in very similar classification accuracy but are various in speeds of convergence. Specifically, GWCS+KD can always reach the highest accuracy after about 2,000 iterations, while FT needs more than 3,500 iterations. This suggests that GWCS+KD is more efficient for network pruning. However, RS+KD does not outperform GWCS+KD and GWCS+FT in the iterations. All of these demonstrate that our GWCS algorithm with KD indeed obtains a promise pruning result.

To summarize, in this study, we propose a novel genetic NAS-based network pruning method to automate the channel-wise network pruning. The main idea is to dynamically select the most informative channels in each layer from the pre-trained network using a multi-stage genetic optimization algorithm. Furthermore, we presented a novel fitness function based on the WCA to evaluate the performance of the pruned network. We conduct large-scale experiments using several public datasets to verify the performance of tested pruning models. The results demonstrate that the proposed GWCS model achieves a more compressed network with a promise classification accuracy than other tested SOTA pruning methods. In the future, we will further evaluate the effectiveness of our model on some mobile devices and employ the proposed model to compress the CNNs for other tasks, such as object detection or image segmentation.

## Data Availability Statement

The original contributions presented in the study are included in the article/supplementary material, further inquiries can be directed to the corresponding author.

## Author Contributions

LC, SG, and XS implemented and optimized the methods and wrote the manuscript. LC, XS, and MS designed the experiment and algorithm. All authors contributed to the article and approved the submitted version.

## Funding

Publication costs are funded by the National Nature Science Foundation of China under grant nos. 61902370, 61802360, and in part by the Chongqing Research Program of Technology Innovation and Application under grants cstc2019jscx-zdztzxX0019.

## Conflict of Interest

The authors declare that the research was conducted in the absence of any commercial or financial relationships that could be construed as a potential conflict of interest.

## Publisher's Note

All claims expressed in this article are solely those of the authors and do not necessarily represent those of their affiliated organizations, or those of the publisher, the editors and the reviewers. Any product that may be evaluated in this article, or claim that may be made by its manufacturer, is not guaranteed or endorsed by the publisher.
